# Development of a High-Density Genetic Map for Muscadine Grape Using a Mapping Population from Selfing of the Perfect-Flowered Vine ‘Dixie’

**DOI:** 10.3390/plants11233231

**Published:** 2022-11-25

**Authors:** Kirill Lytkin, Vasily Nosulchak, Magamedgusein Agakhanov, Elena Matveikina, Ekaterina Lushchay, Dmitry Karzhaev, Evgenii Raines, Irina Vasylyk, Nataliya Rybachenko, Elizaveta Grigoreva, Vladimir Volkov, Vladimir Volynkin, Laurent Gentzbittel, Elena Potokina

**Affiliations:** 1 All-Russian National Research Institute of Viticulture and Winemaking ‘Magarach’ RAS, Yalta 298600, Russia; 2 Institute of Forest and Natural Resources Management, Saint Petersburg State Forest Technical University, St. Petersburg 194021, Russia; 3 N.I. Vavilov All-Russian Institute of Plant Genetic Resources (VIR), St. Petersburg 190031, Russia; 4 Information Technologies and Programming Faculty, ITMO University, St. Petersburg 197101, Russia; 5 Skolkovo Institute of Science and Technology, Moscow 121205, Russia

**Keywords:** high-density linkage map, *Muscadinia rotundifolia* cv. Dixie, restriction site-associated DNA sequencing, single nucleotide polymorphism, QTL mapping, seedling morphological traits

## Abstract

Intraspecific diversity of the immune grape *Muscadinia rotundifolia* Michaux. can serve as a rich source of valuable resistance loci to the most widespread pathogens and pests of grapevine. While only one *Run1/Rpg1* resistance locus has been introgressed from *M. rotundifolia* to the *Vitis vinifera* gene pool, a number of other genes conferring resistance to powdery mildew and downy mildew have been identified in various *Muscadinia* cultivars. A larger introduction of *Muscadinia* varieties to the European continent would greatly facilitate experiments of interspecific crosses as well as stimulate biotechnological efforts to overcome the main barrier to F1 fertility caused by the differences in chromosome number. For the successful introduction of *Muscadinia* into the new European environment, it is necessary to overcome the difficulties associated with the physiological characteristics of the species, such as insufficient cold tolerance and very late fruit ripening. To facilitate the further discovery of valuable loci in *Muscadinia* and their transfer to grapevine breeding programs, we constructed a high-density linkage map using an S1 mapping population obtained from the self-pollination of *M. rotundifolia* cv. Dixie maintained on the southern coast of Crimea. Using ddRADseq, 3730 SNPs were ordered across 20 linkage groups spanning 2753.6 cM of the total map length. No segregation in resistance to diseases and pests was observed among the ‘Dixie’ S1 population, suggesting the presence of homozygous non-segregating resistant loci in the genetic background of ‘Dixie’. Markers with high segregation distortion showed a bias towards chromosomal intervals on linkage groups 10 and 20, where loci affecting the survival of ‘Dixie’ S1 progeny may be localized. QTLs with significant additive and dominance effects were discovered on LG14 and LG18, affecting the morphological traits associated with the vigor of growth and adaptability of young *Muscadinia* vines in the conditions of Crimea.

## 1. Introduction

The great interest of European grapevine breeders in the North American *Muscadinia* species is explained by the potential possibility to introgress disease and pest resistance loci into the new grapevine varieties they create. *M. rotundifolia* exhibit strong resistance to root and leaf forms of grape phylloxera, the bacterial causal agent of Pierce’s disease, rootknot nematodes, the nematode vector of grapevine fanleaf virus, anthracnose, and the most widespread grape fungal diseases powdery and downy mildew [1]. However, the introgression of the desired loci is complicated by the different number of chromosomes in the two subgenera of *Vitis*: *Muscadinia* (2n = 40) and *Euvitis* (2n = 38), resulting in sterile F1 from interspecific crosses. Even if this complication is occasionally overcome, the rarely fertile F1 usually inherit a very poor rooting ability from *M. rotundifolia* which limits their use as rootstocks [2]. In addition, the introgression of a resistance gene often results in the linkage drag of undesired traits from *M. rotundifolia* that may remain even after successive cycles of backcrossing [3].

Recent inspiring advances in biotechnology, including gene and genome editing, provide new opportunities to develop resistant forms of cultivated grapes without introducing alien genetic material into the grape plant. From this perspective, knowledge of the genetic factors that determine the durable resistance of *Muscadinia* varieties becomes vital for grape breeders. 

The efficiency of grape breeding programs can be greatly improved by marker-assisted selection, which in turn requires the creation of extensive sets of molecular markers and their linkage mapping. The first genetic maps for *M. rotundifolia* were based on SSR markers and used either the offspring from a cross of two different *M. rotundifolia* varieties or the progenies obtained from the self-pollination of a single hermaphroditic plant as a mapping population. For the first case, a pseudo-test cross-mapping strategy was applied [4], and two separate maps for each parent (cv. Fry and cv. Trayshed) plus a consensus map with a total distance of 1088 centiMorgan (cM) were developed [1]. In the latter case, the mapping population produced from the selfing (S1) of *M. rotundifolia* cv. Regale was used to obtain a linkage map covering 948 cM on 20 linkage groups [3] using 178 SSR markers. 

Although early molecular research in muscadine linkage mapping has been mostly focused on the identification of disease resistance loci, it also provided the first insight into the evolution and phylogeny of the *Vitis* species. Perfect collinearity between the genetic map of *M. rotundifolia* and *V. vinifera* reference map was reported [1]. With a linkage map built for *M. rotundifolia* cv. Regale, *V. vinifera* chromosome 7 (LG7) was first found to correspond to two separate LG7 and LG20 in the *M. rotundifolia* map, and *M. rotundifolia* LG20 was found to correspond to the bottom part of *V. vinifera* LG7 [3]. The hypothesis was further supported by the results of high-density linkage muscadine mapping based on genotyping-by-sequencing technology. The highly saturated genetic maps that were constructed for crosses BlackBeauty’ × ‘Nesbitt’ and ‘Supreme’ × ‘Nesbitt’ resulted in 1244 and 2069 markers spanning 20 chromosomes [5]. The tremendously increased number of markers allowed for the defining of the precise location where *V. vinifera* chromosome 7 is split into two chromosomes in muscadine. In addition, the flower sex and berry color loci had first been mapped in the *Muscadinia* genome.

The new era of *Muscadinia* genome research began with the release of two chromosome-level genome sequence assemblies for cv. Trayshed [6] and cv. Noble [7]. The smaller genome size of *M. rotundifolia* compared to *V. vinifera* and highly conserved gene synteny had been established. With the reference genome available, it became possible to explore each chromosome interval where QTLs affecting a trait of interest were detected. As an example, numerous quantitative trait nucleotides (QTNs) were identified by an association analysis of 12 berry-related traits of *Muscadinia* grapes, including fruit color-related traits, length, width, weight per berry, and berry firmness. For the trait ‘berry color’, annotated genes between the two flanking markers of the QTN were investigated in the *Muscadinia* genome. Among 29 identified genes, a candidate gene encoding glutathione-S-transferase was detected associated with the anthocyanin biosynthesis pathway [7].

Linkage mapping efforts in *Muscadinia* also revealed an important observation: Different muscadine varieties provide various genetic sources of desirable traits, e.g., resistance to pathogens. For example, the most-used locus of resistance to powdery mildew *Run1* on LG12 was introgressed to the *V. vinifera* genome from the NC6-15 interspecific hybrid. This hybrid NC6-15, in turn, inherited its resistance from G52, a cross between the *M. rotundifolia* cultivars ‘Thomas’ and ‘Hope’ [8]. Other strong loci of resistance for the same pathogen were localized on LG18 of cv. Magnolia (*Run2.1*) and cv. Trayshed (*Run2.2*). One major QTL for resistance to grapevine powdery mildew, named *Ren5,* was located on linkage group LG14 of the genetic map constructed with the mapping population produced from the selfing (S1) of *M. rotundifolia* cv. Regale [3]. Thus, the diversity of *M. rotundifolia* varieties provides a rich source of various valuable loci, and their discovery is often a matter of QTL mapping using progenies from the crossing of different *M. rotundifolia* varieties or even progenies obtained from the selfing of a single hermaphrodite muscadine plant.

In the present paper, we report the construction of a high-density linkage map of *M. rotundifolia* cv. Dixie, a perfect-flowered (hermaphrodite) muscadine vine. To our knowledge, the variety ‘Dixie’ is the only cultivated representative of the *Muscadinia rotundifolia* species in the North Caucasus and Crimea Peninsula. ‘Dixie’ was introduced to the VIR germplasm collection in 1990 as a single plant. Due to its hermaphrodite flowers, the ‘Dixie’ vine produces viable seeds that provide the opportunity to establish an S1 mapping population and explore the phenotypic variability of the muscadine vine under new conditions. 

## 2. Results

### 2.1. Performance of the North American Muscadine Grape ‘Dixie’ in the Southwestern Part of the North Caucasus

The muscadine grape ‘Dixie’ was introduced to the Krymsk branch of the VIR germplasm collection (Krasnodar area, the southwestern part of the North Caucasus) in 1990 along with the other 33 varieties of *M. rotundifolia*, one cutting for each variety. Rooting muscadines from hardwood cuttings is a well-known challenge for muscadine breeding [9], so only the ‘Dixie’ vine was successfully rooted. 

‘Dixie’ muscadine vine is a hermaphrodite plant, presumably derived from the H2 source of hermaphroditism. The last one was developed by crossing the female muscadine cultivar ‘Scuppernong’ and the muscadine pollinizer ‘New Smyrna’ [5]. Both those varieties are in the pedigree list of ‘Dixie’ (Supplementary Materials, Figure S1). Seeds were obtained from the self-pollination of the ‘Dixie’ single plant at the Krymsk branch of VIR. Cross-pollination with any other *Vitis* accessions from the grapevine germplasm collection was excluded due to different chromosome numbers of *Muscadinia* (2n = 40) and *Euvitis* (2n = 38) subgenera.

Although, in general, the climatic conditions of the Krasnodar area are favorable for viticulture, in some years, the minimum winter temperatures in Krymsk can drop to −15 °C. Due to the low frost resistance, the ‘Dixie’ plant could not fully recover after several harsh winters, so its fruiting in Krymsk was recorded only 9 times over 18 years of observation. 

**Figure 1:**
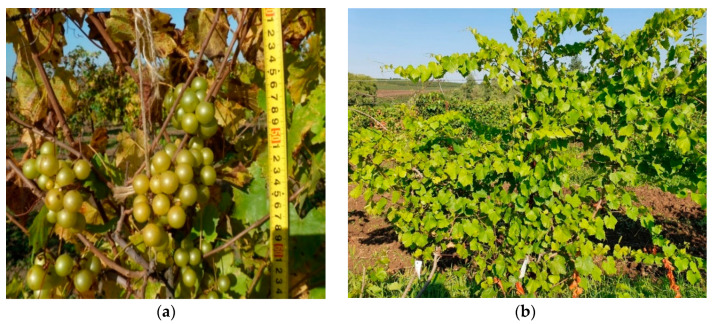
*Muscadinia rotundifolia* cv. Dixie at the experimental vineyard of the Krymsk Experimental Station of VIR (the southwestern part of the North Caucasus) (photo by V.A. Nosulchak): (**a**) fruiting in 2021; (**b**) plant habit of a twenty-year-old S1 progeny produced from selfing of ‘Dixie’ plant.

From the seeds of the first fruiting in 2003, more than 200 S1 seedlings were grown, of which 28 were preserved by 2021. Some of them entered fruiting and produce seeds. The last four years, possibly due to climate warming and, consecutively, mild winters, the ‘Dixie’ vine and its S1 progenies recovered from freezing quickly and produced seeds regularly (Figure 1). 

The regular field evaluations of ‘Dixie’ and its S1 progenies started in 2018 and have provided some valuable observations. Under the climatic conditions of the North Caucasus, the muscadine grape ‘Dixie’ was not affected by fungal pathogens (e.g., powdery and downy mildew) or phylloxera. Segregation in resistance to diseases and pests among the ‘Dixie’ S1 population was not observed. This suggests the presence of homozygous non-segregating factors in the genetic background of ‘Dixie’ that provide complete resistance to the main pathogens that endanger viticulture throughout Europe. 

The major drawback of the muscadine vine in Crimea and the North Caucasus is low frost resistance and very late ripening of berries. The winter hardiness of cv. Dixie depends very much on the lowest temperatures of the particular year. In 2018, with the lowest winter temperature of −8 °C, up to 80% of ‘Dixie’ S1 progeny quickly recovered and delivered ripe berries of consumable quality, while in 2021 (minimum winter temperature −15.6 °C), only 20% of S1 plants were able to produce ripe berries.

With the exception of bud burst, the other phases of development (flowering and ripening) occurred in muscadine vines much later than in the *V. vinifera* varieties. The beginning of the bud burst took place in May simultaneously with the beginning of vegetation in the *V. vinifera* varieties and *Vitis* interspecific hybrids. There were no significant differences between S1 progenies for this phenological trait.

The onset of flowering was observed at the end of June–July, 20–30 days later than *V. vinifera* accessions. The difference between S1 progeny and the mother plant ‘Dixie’ was not substantial (up to 9 days). All S1 progenies had a bisexual flower type like the parental genotype.

In terms of the start of ripening of berries, ‘Dixie’ and its S1 progenies were late compared to the latest varieties of *V. vinifera* maintained in the Krymsk germplasm collection, such as Agadai, Granatovyi, Karaburnu, Moldova, Pamjati Verderevskogo, Rkatiteli, and Saperavi. As a result, only single S1 plants were able to produce berries with a sugar content corresponding to the level of consumer ripeness (17%). Remarkably, large segregation was observed between S1 progenies by the date of ripening (with a difference of up to 30 days); some of them were ahead of the parent ‘Dixie’ by 12 days. Thus, selection for early ripening among the muscadine vines can be promising in the North Caucasus. Significant variability was also observed among S1 progenies in terms of the power of plant development and fruiting level. The two most vigorous and productive ’Dixie’ S1 offspring were selected as valuable materials for further muscadine vine breeding.

**Figure 2:**
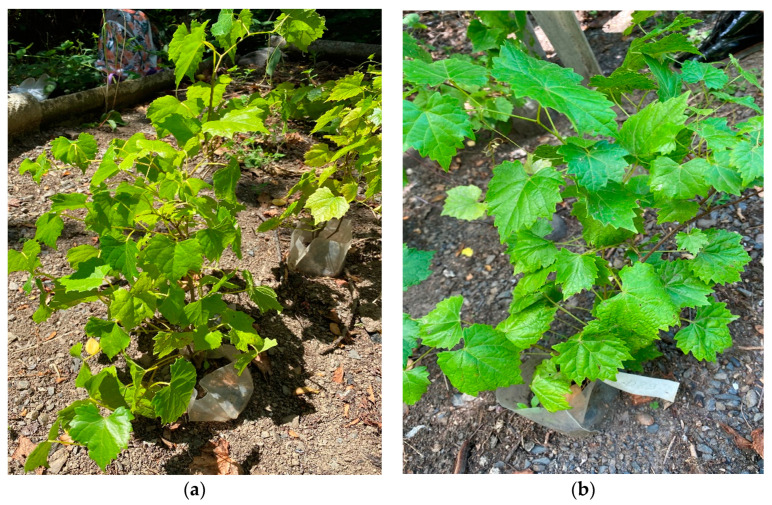
Three-year-old rooted seedlings obtained from selfing (S1) of *M. rotundifolia* cv. Dixie and grown in the experimental vineyard of the Institute of Viticulture and Winemaking ‘Magarach’ (Crimea): (**a**,**b**) vines with varying morphological characteristics.

The brief review of the performance of the muscadine variety ‘Dixie’ in the North Caucasus was based on the observation of its 28 twenty-year-old S1 progenies. In order to increase the size of the ‘Dixie’ S1 population and make it suitable for genetic studies aimed at developing molecular markers, a new batch of seeds was produced from the selfing of *M. rotundifolia* cv. Dixie and collected in 2019. The seeds were germinated, and in 2020, one-year-old seedlings were transferred to the Institute of Viticulture and Winemaking ‘Magarach’ (Crimea). To date, 84 plants from ‘Dixie’ S1 progeny have been successfully maintained in the experimental vineyard of the ‘Magarach’ Institute. This newly established ‘Dixie’ S1 population of three-year-old rooted vines (Figure 2) was genotyped by NGS to identify SNP markers and generate a high-density linkage map.

### 2.2. Development of SNP Markers Based on NGS Data

ddRAD-seq libraries were constructed for the parent ‘Dixie’ in two replications and 84 progenies from ‘Dixie’ selfing (S1 mapping population). In total, 179,554,345 reads were obtained for the 84 progenies and 15,537,401 for cv. Dixie using the Illumina HiSeq2500 sequencing platform.

After quality control, raw reads were aligned to each of the two available versions of the *M. rotundifolia* genome assembly: cv. Trayshed [6] and cv. Noble [7]. Additionally, reads were mapped to the *V. vinifera* PN40024 genome [10]. As the result, 56% of reads were successfully aligned on the *V. vinifera* genome while 76% and 72% of reads were well mapped on the genome of *M. rotundifolia*: cv. Trayshed and cv. Noble, correspondingly. In this step, we estimated how many polymorphic loci were identified as heterozygous for cv. Dixie in each of the alignments. Table 1 shows the estimated heterozygosity of cv. Dixie slightly varied around 0.20, which is in line with the previous report about heterozygosity ranging from 0.26 to 0.39 across the 15 wild and five cultivated *Muscadinia* populations [11].

Next, SNPs were filtered by the maximum missing calls observed among 84 progenies. After filtering by the stringent criteria MISS = 0.90, VCFtools kept in analysis 34,904 out of a possible 1,783,373 sites (Table 1). 

Since only heterozygous loci of cv. Dixie were considered, a chi-square goodness-of-fit test was applied to remove loci that deviated from the expected 1:2:1 segregating pattern in the ‘Dixie’ S1 population. Thus, 3734 SNPs with no more than 10% of missing data significantly matching a segregation ratio of 1:2:1 (*p* <0.05) were saved for mapping (Supplementary Materials, Table S1).

### 2.3. Construction of the Muscadinia rotundifolia ‘Dixie’ Linkage Map

A total of 3734 high-quality SNPs, heterozygous in the ‘Dixie’ genome, and showing a 1:2:1 segregating pattern in the ‘Dixie’ S1 population were first assigned to 20 linkage groups based on their physical positions on the cv. Noble reference genome. Next, the SNPs were subjected to linear ordering within each LG by maximizing the likelihood of the data with Lep-MAP3 software. The resulting genetic map of cv. Dixie contained 3730 markers spanning 2753.6 cM (Table 2 and Supplementary Materials, Table S2). The total length of the constructed linkage map was comparable with the previously reported length for the highly saturated genetic map for *Muscadinia* varieties. For example, the consensus map created from the ‘Black Beauty’ × ‘Nesbitt’ and ‘Supreme’ × ‘Nesbitt’ F1 mapping populations was estimated as 2164 cM [5]. In a line with this study, LG14 (169.9 cM) was the longest linkage group (Table 2). The shortest chromosome in cv. Dixie linkage map was LG11, while for *M. rotundifolia* ‘Black Beauty’, ‘Nesbitt’, and ‘Supreme’ cultivars, LG8 was indicated as the shortest. 

**Figure 3:**
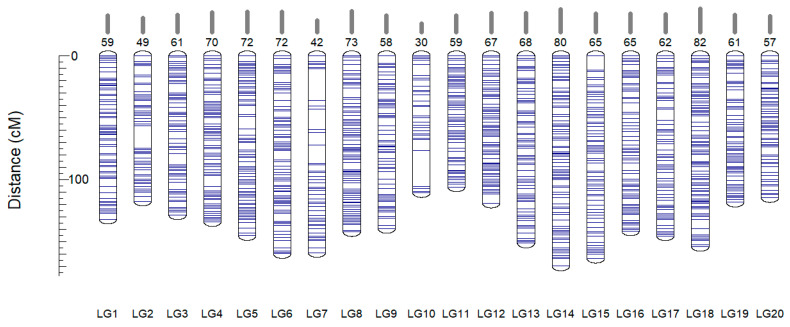
Linkage map of *Muscadinia rotundifolia* cv. Dixie developed using a mapping population derived from self-pollination of a hermaphrodite cv. Dixie. The number of segregating markers per chromosome is indicated above the corresponding LG.

The average genetic interval between the nearest markers of the map varied from 1.8 to 3.8 cM depending on the linkage group. The largest gaps were observed for LG10 (29 cM), LG7 (25 cM), and LG2 (18 cM) (Table 2, Figure 3). The lack of markers spanning those chromosome intervals can be explained by random factors, but genomic variations between cv. Dixie and cv. Noble can also be assumed. At least ten partial inversions, three translocations, and five inversions with translocation were reported between two *Muscadinia* cultivars cv. Noble and cv. Trayshed [7]. According to the report, LG 2,3,10, 12, and 15 are the chromosomes where the rearranges were detected. 

### 2.4. Correlation between Genetic and Physical Distances on the Muscadinia rotundifolia ‘Dixie’ Linkage Map

**Figure 4:**
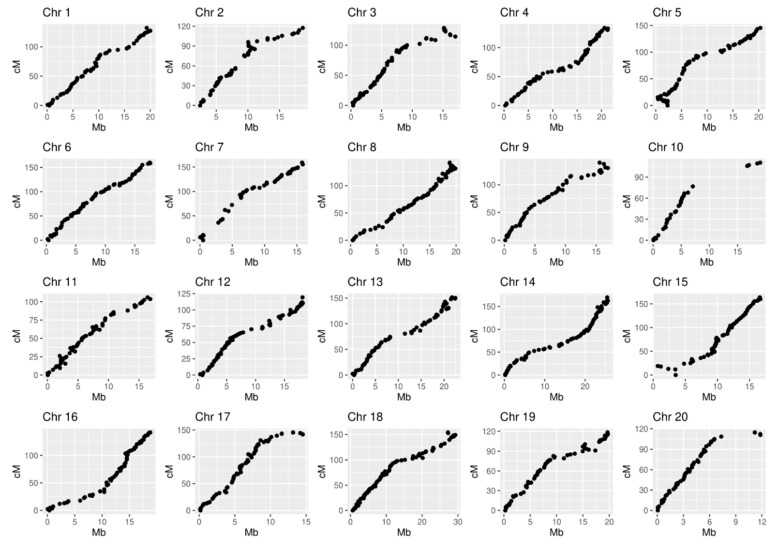
Collinearity revealed between the genetic distances on the *M. rotundifolia* ‘Dixie’ (cM) linkage map and the physical position (Mb) of the SNP in the *M. rotundifolia* cv. ‘Noble’ genome.

The linear order of SNP markers along each chromosome was determined based on the recombination frequency observed within the S1 ‘Dixie’ mapping population. The collinearity of genetic and physical distances was evaluated for 20 linkage groups (Figure 4). The Pearson correlation coefficient varied from 0.92 (LG10) to 0.99 (LG6) with an average value of 0.97. Although most chromosomes yielded a good performance, small inversions on LG3 (114–128 cM), LG5 (0–22 cM) and possible translocation on LG15 (0–23 cM) were detected (Supplementary Materials, Figure S2). 

A major discrepancy was observed for LG10 and LG20, probably due to the largest gaps detected for the two chromosomes (Figure 4). The detected mismatches between genetic and physical distances can occur due to sequencing-related genotyping errors [12]. On the other hand, the strategy of eliminating markers with high segregation distortion (SD) can also lead to blank areas on the map since SD can be caused by loci affecting progeny survival, gamete function, or meiosis. Indeed, 19.6% of original ‘Dixie’ S1 progeny did not survive after field planting, and a genetic reason for this can be assumed. To consider whether the unmapped regions are due to the lack of SNPs filtered by SD criteria, we compared the physical distributions of 34,904 high-quality SNPs (Table 1), and 3734 of them that passed the chi-square fit test (Supplementary Materials, Figure S3). As shown in Figure S3, the character of the distribution of SNP markers along the chromosome before and after filtering according to the SD criteria is similar with the exception of LG20 and, especially, LG10. We assume that the interval between SCP092935.1_7063939 and SCP092935.1_16723504 on LG10 as well as the interval SCP092945.1_7337634-SCP092945.1_10853675 on LG20 may contain loci affecting the survival of progeny obtained from ‘Dixie’ self-pollination. 

### 2.5. Assessment of the ‘Dixie’ S1 Population for Resistance to Powdery and Downy Mildew and the Variation of Morphological Traits 

Combining a high-density linkage map generated using an *M. rotundifolia* cv. Dixie S1 population and field evaluation data, we attempted to find loci affecting valuable agro-biological traits of this muscadine vine, which can be revealed by their segregation in the ‘Dixie’ S1 progeny.

First, the established S1 mapping population of *M. rotundifolia* cv. Dixie was evaluated for resistance to powdery mildew (oidium, *Erysiphe necator*) and downy mildew (*Plasmopara viticola*) in a laboratory experiment. Fragments of shoots with three leaves were artificially infected with an aqueous suspension of oidium conidia washed off from infected grapes collected on the southern coast of Crimea. Additionally, a dry powder of oidium conidia was applied to infect the leaves. Inoculated leaves were scored for the general level of resistance (OIV452) three, seven, ten, and fourteen days post-inoculation, and no signs of infection were detected.

Similarly, in experiments with the artificial treatment of ‘Dixie’ S1 progeny with downy mildew conidia (see Methods), no infection was observed three, seven, ten, and fourteen days after inoculation. In the field, we also did not observe signs of infection with mildew and oidium on ‘Dixie’ seedlings. Thus, we concluded that ‘Dixie’ muscadine may have homozygous non-segregating loci conferring complete resistance to the fungal pathogens.

Given the significant variation of the ‘Dixie’ S1 seedlings by their ability to overcome winter frosts and successfully survive in a new stressful environment, we evaluated a number of morphological traits that may reflect the vigor and adaptability of each particular seedling. As follows from Table 3, six evaluated traits describing the overall morphology and performance of each ‘Dixie’ S1 seedling show a significant correlation with each other. 

To identify loci affecting the variation of the morphological traits, QTL analysis was performed. The S1 mapping population in which each marker has the same 1AA:2AB:1BB segregation pattern can be considered an F2 population; however, the linkage phases between marker alleles need to be estimated. This estimation was performed according to Wu et al. [13] using the onemap: map function implemented in OneMap software [14]. The function estimates the multipoint log-likelihood, linkage phases, and recombination frequencies for a sequence of markers in a given order. As a result, the parent and progeny phased haplotypes estimated by OneMap hidden Markov model (HMM) were generated (Supplementary Materials, Table S2).

Composite interval mapping (CIM) was then performed to identify both additive and dominant effects of SNP markers on the variability of six morphological traits. The obtained threshold values for LOD scores using 1000 permutations ranged from 3.30 to 3.33 among six traits and were used to declare the presence of QTLs. Significant QTLs (*p* < 0.05) were detected for average shoot length, average internode length, and average leaf diameter (Table 4).

**Figure 5:**
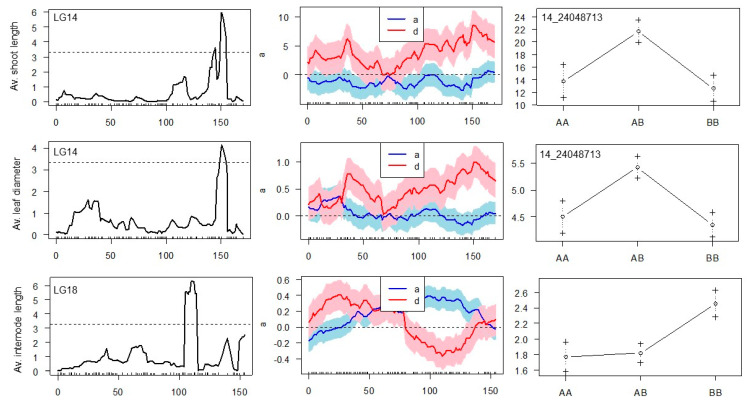
LOD profiles, additive, and dominance effect of the QTL Composite Interval Mapping for morphological traits assessed for ‘Dixie’ S1 progenies: top panel-average length of shoots per plant, middle panel-average leaf diameter, bottom panel-average internode length. Two colored lines in each of vertical middle plot represent the additive (a) and dominance (d) effects, colored regions indicate ± 1SE. Phenotype means (°) and error bars (+) are plotted at ±1 SE for each genotype group (AA, AB and BB). QTL peak mapped on LG14 coincided with the marker SNP_24048713 (150.55 cM), QTL peak on LG18 (116.8 cM) co-segregated with five SNP markers.

A CIM algorithm located the single QTL on the LG14 in the same position of 150.55 cM for ‘average length of shoots’ and ‘average leaf diameter’, explaining 27.8% and 20.2% of the observed phenotype variation, respectively. This QTL demonstrates a clear dominance effect arising from interactions between alleles at the same locus (Figure 5). 

‘Average length of shoots’ and ‘average leaf diameter’ can be considered major characteristics reflecting the viability of ‘Dixie’ S1 seedlings, and they were found to segregate depending on the QTL with the peak, where the SNP marker with the physical position 24,048,713 on the genome of cv. Noble was mapped. Reads of cv. Noble genome overlapping 142 bp in length and embedding the SNP_24048713 were aligned to the annotated cv. Trayshed genome, in which their physical position corresponds to the interval 21,638,800–21,638,940 of the assembly. This is approximately 1.04 kb apart from the 21,639,976…21,669,459 functional sequence of cv. Trayshed, which is a putative homolog of the LOC100262888 coding sequence in the genome of *V. vinifera* cv. Pinot Noir (PRJEA18785). The gene encodes inositol hexakisphosphate and diphosphoinositol-pentakisphosphate kinase 1, which are involved in the biosynthesis of inositol pyrophosphates (PP-InsPs), the “high-energy” intracellular signaling molecules with suggested roles in bioenergetic homeostasis and inorganic phosphate (Pi) sensing in plant cells [15].

QTL with the highest LOD score and a significant additive effect was mapped on LG18 for the trait ‘average internode length’, explaining 29% of phenotypic variation (Figure 5). Five SNPs were found co-segregating with this QTL and spanning a physical distance of 539 kb (Table 4). The additive effect of the QTL appears due to a significant difference in the mean phenotypes of the ‘AA’ and ‘BB’ genotype groups of S1 progenies (*p* < 0.001). In the example for SNP 18_21141408, which is heterozygous (T/C) in the ‘Dixie’ genome, the “AA” progeny group carries a homozygous reference allele (T/T), while the “BB” progeny group has a homozygous locus with an alternative allele (C/C). It seems that the presence of the reference allele (T/T or T/C) negatively affects the elongation of the internodes of ‘Dixie’ S1 progenies.

The identified SNPs possibly ‘highlight’ the LD blocks on the ‘Dixie’ genetic map, which may harbor functional sequences affecting fitness-related traits in ‘Dixie’ S1 progeny. In the *Muscadinia* genome, linkage disequilibrium (LD) between adjacent markers averaged as r^2^ = 0.36, and the distance over which LD decayed to half of the initial value was ∼2.3 Mb [7]. Thus, a detailed study of the polymorphism of this neighboring coding sequence between ‘Dixie’ offspring with different fitness-related traits may provide a possible marker for the selection of genotypes with higher adaptability.

## 3. Discussion

The intraspecific diversity of *Muscadinia rotundifolia* serves as the base of the muscadine grape industry of the southeastern USA, which produces fresh fruit, juices, and wine [1]. The first public muscadine breeding programs have been initiated in the last century and succeeded in the development of perfect-flowered muscadine cultivars as well as increasing vine yield, berry size, and fresh market berry quality [16]. Nowadays, significant muscadine breeding efforts are being made at the southeastern USA’s universities and horticultural research centers [11]. 

*M. rotundifolia* was introduced to Europe at the end of the 19th century but did not arouse real interest among European grape growers due to limited success in the acclimatization of the vine species [17]. Cold tolerance is one of the issues for muscadine breeding programs even in Arkansas because of its location near the northern limits of the *M. rotundifolia* distribution area [11].

Due to some successful attempts to obtain fertile *M. rotundifolia* × *V. vinifera* interspecific hybrids (e.g., NC6-15), loci of resistance to pathogens *Erysiphe necator* (*Run1*) and *Plasmopara viticola* (*Rpv1*) were transferred into the *V. vinifera* European gene pool [17]. Although several other genetic sources of resistance to powdery mildew and downy mildew have been identified in the past few decades among the diversity of *Muscadinia* cultivars (see [1,3]), *RUN1/RPV1* is still the only resistance locus that has been introduced to *V. vinifera* through grape breeding programs [7].

A larger introduction of the *Muscadinia* varieties on the European continent would greatly facilitate experiments of interspecific crosses as well as stimulate biotechnological efforts to overcome the main barrier to F1 fertility caused by the differences in chromosome number. However, for the successful introduction of *Muscadinia* into the new European environment, it is necessary to overcome the difficulties associated with the physiological characteristics of the species, such as poor rooting of cuttings, insufficient cold tolerance, and very late fruit ripening. Our study on the propagation and evaluation of *Muscadinia* cv. Dixie proves that these limitations can be overcome and demonstrates the real possibility of cultivating *Muscadinia* varieties in Crimea and the North Caucasus. 

The complete immunity of *Muscadinia* cv. Dixie to local Crimean and North Caucasian strains of oidium and mildew pathogens was observed in our study. It was reported that powdery mildew can respond rapidly to host-based selection pressure and generate isolates that can overcome *M. rotundifolia’s* resistance [8]. For example, cv. Magnolia is reported to be susceptible in North Carolina, but it has never shown symptoms in California [8]. Results of field and laboratory observations on the resistance of cv. Dixie outside the American continent testifies to the presence in its genome of loci that confer strong resistance to broad-spectrum strains of the pathogens. Although we could not identify the loci responsible for the immunity of cv. Dixie to powdery and downy mildew due to the absence of segregation in S1 progeny, cv. Dixie can still be considered a valuable donor of resistance genes for interspecific crosses.

It can be assumed that the resistance of cv. Dixie has the same origin as NC6-15, which in turn inherited its resistance from G52, a cross between *M. rotundifolia* cv. Thomas and cv. Hope. Besides these two varieties, the pedigree list of cv. Dixie contains other possible sources of resistance: *Muscadinia* varieties Scuppernong, New Smirna, Stanford, Latham, Burgaw, Willard, Topsail, Lucida, Wallace, Irene, at least two *Muscadinia* unknown landraces, and another American *Vitis* species—*V. lincecurnii* (Supplementary Materials, Figure S1).

DNA-based molecular markers play an important role in grape cultivar identification and pedigree verification, which are essential to facilitate breeding programs. Molecular databases established with SSR markers have been developed as tools for authentic identification of *V. vinifera* varieties, allowing to find duplicates and misnames [18,19,20], identify population structure within the supported diversity, and build a core collection [21]. Accordingly, 81 unique accessions have recently been genotyped in *Muscadinia* using 20 SSRs from 13 linkage groups, and an SSR-based molecular database has been developed to facilitate cultivar identification and muscadine germplasm management [22].

Both SNP and SSR markers have proven effective in assessing genetic diversity and population structure in large germplasm collections of grapevines [23]. In this study, we generated 34,904 high-quality SNPs that were identified by aligning homologous sequences of cv. Dixie and cv. Noble (Table 1). Notably, this number of SNPs is six times greater than the number of polymorphic SNPs identified between cv. Dixie and cv. Trayshed (5490), which is consistent with reports of close genetic similarity between the two varieties revealed by clustering their genetic profiles obtained by the rhAmpSeq method [11]. Out of 34,904 SNPs detected, 3730 were used to construct a high-density linkage map of *M. rotundifolia* cv. Dixie that will facilitate the involvement of cv. Dixie in grape breeding programs. Only molecular markers showing a 1:2:1 segregation pattern, heterozygous in the parent ‘Dixie,’ were employed for mapping. Remarkably, markers with high segregation distortion, which were excluded from mapping, showed a bias towards chromosomal intervals on linkage groups 10 and 20, where loci affecting the survival of ‘Dixie’ S1 progeny may be localized. 

The obtained S1 mapping population emulates F2 (all markers with segregation pattern 1:2:1); however, the linkage phases between the markers are unknown. Although marker ordering and recombination fraction estimation are still possible using heterozygous markers with unknown linkage phase, QTL analysis will be limited as it only detects loci with a dominance effect, reflecting a significant phenotypic difference between homozygous and heterozygous progenies. A new methodology has been proposed for the simultaneous estimation of linkage and linkage phase configurations over all linked markers in a full-sib family in outcrossing species based on a maximum likelihood approach [13]. OneMap software running this algorithm was used to predict the linkage phase between markers. As a result, QTL analysis revealed loci with both dominance and additive effects.

We report a QTL with a highly significant dominance effect discovered on LG14 in the position of 150.5 cM, affecting the strength of growth and development of muscadine seedlings in the early stages of their ontogenesis. The QTL explained up to 28% of the observed phenotypic variation, and, consequently, significantly contributed to the total genetic variance for these morphological traits. The putative candidate gene located ~1.03 kb downstream of the SNP_24048713 may encode inositol phosphate kinase, which transfers phosphates to an inositol ring. In *Arabidopsis,* the enzymes hexakisphosphate and diphosphoinositol-pentakisphosphate kinase 1 are involved in the biosynthesis of inositol pyrophosphate signaling molecules, which are of great agronomic importance as they can control complex responses to the limited nutrient phosphate [15]. 

Another group of SNP markers within the QTL peak on LG18 was discovered for the trait ‘average internode length’. These SNPs cover a physical distance of ~500 kb and build a possible LD block, where a certain haplotype negatively affects the internode elongation of ‘Dixie’ S1 seedlings. BLAST analysis of the sequences embedding the discovered SNPs was performed against the genome of *V. vinifera* cv. Pinot Noir. Among five marker loci detected, two neighboring SNPs (18_20602071 and 18_20601991) fall into the second exon of the gene LOC100260625 encoding TMV (tobacco mosaic virus) resistance protein N, which triggers a defense system, including the hypersensitive response restricting the pathogen growth. The sequence containing two other SNPs 18_21074021 and 18_21074082 showed homology to the intron region of gene LOC100251493 encoding DExH-box ATP-dependent RNA helicase. In plants, DExD/H-box RNA helicases have been demonstrated to be associated with plant development and abiotic stress tolerance through their functions in modulating pre-rRNA processing [24]. The sequence surrounding SNP 18_21141408 showed similarity to the second intron of the LOC100246359 coding sequence, whose predicted function is “receptor homology region, transmembrane domain- and RING domain-containing protein 2”. In *Arabidopsis,* the protein belongs to the family of type I transmembrane proteins, the Receptor Membrane RING-H2 (RMRs); these are specific transmembrane cargo-receptors participating in the sorting of soluble proteins to vacuoles, involved in essential functions related to plants growth, development, and response to environmental stresses [25].

The constructed high-density genetic map for *Muscadinia rotundifolia* cv. Dixie can be further employed for mapping QTLs affecting agronomical traits that will appear as ‘Dixie’ S1 seedlings grow and proceed to fruiting. The S1 population segregating for cold tolerance and productivity can be involved in breeding programs as a new source of valuable traits. 

## 4. Materials and Methods

### 4.1. Plant Material

The plant of *M. rotundifolia* cv. Dixie was grown from a single cutting obtained from the US in 1999 in a program of plant genetic resources exchange between gene banks. Since that time, the rooted vine of the ‘Dixie’ variety has been kept in the *Vitis* germplasm collection of the Krymsk Experimental Station of VIR (44°56′00″, 38°00′00″).

One-year-old seedlings grown from seeds obtained from self-pollination of *M. rotundifolia* cv. Dixie were transferred to the Magarach Institute of Viticulture and Winemaking (Yalta, Crimea) in December 2020. These plants were transplanted into separate plastic bags for seedlings in a soil–peat mixture and numbered. To adapt the seedlings to the growing conditions on the southern coast of Crimea, the bags with seedlings were placed in a row with a fogging machine, where regular watering and plant care were carried out. The next two years, the seedlings grew in natural conditions without shading. As a result, 84 three-year-old seedlings that successfully survived the new environment were used for this study. 

### 4.2. Inoculation of ‘Dixie’ S1 Seedlings with Conidia of Powdery and Downy Mildew

When studying resistance of ‘Dixie’ S1 seedlings to powdery mildew and downy mildew, inoculation was carried out with Crimean local isolates of pathogens *Erysiphe necator* and *Plasmopara viticola* collected from the southern coast of Crimea. For inoculation, we used a method that has proven itself well in our previous studies and was described earlier [26,27]. In particular, for a laboratory experiment with inoculation by powdery mildew isolate, a segment of a shoot with three leaves was taken from each plant in order to keep the leaves fresh as long as possible since oidium is an obligate parasite and develops on living tissue. After collecting in the field, the shoot segments were immediately taken into a plastic bag and numbered. In the laboratory, shoot segments were laid out in large square Petri dishes on wet filter paper, and the lower end of the shoot was wrapped with cotton wool soaked in water to prevent drying.

To prepare the powdery mildew isolate for subsequent inoculation, a flush was made from grape bunches affected by oidium harvested in Livadia (the southern coast of Crimea) (Supplementary Materials Figure S4). The resulting isolate solution was checked, and the inoculation was carried out. When there were no signs of infection in three days, additional affected grape bunches were collected and dry conidia of oidium from the affected berries were applied with a brush on the same leaves, plus an infected berry was placed next to each shoot. 

For inoculation with conidia of downy mildew, the same procedure was followed, except that, after inoculation, the Petri dishes were wrapped in a black plastic bag until assessed for infection. Infected plant material used to prepare the inoculum was collected in the Balaklava region (the southern coast of Crimea) and placed in a desiccator before the experiment to increase sporulation on the underside of the leaves. Two days later, an inoculation experiment was carried out.

### 4.3. Genotyping Using ddRADseq

For DNA isolation, fresh leaves were collected from each plant in the experimental vineyard of the Magarach Institute of Viticulture and Winemaking (Yalta, Crimea) and frozen in liquid nitrogen. DNA was isolated with the protocol we previously described [28].

The original plant ‘Dixie’ and S1 progenies from its selfing were genotyped using the double-digest ddRADseq approach [29]. The description of the procedure performed, e.g., construction and sequencing of ddRAD libraries, has been published previously [28] (https://www.mdpi.com/2223-7747/10/6/1215, (accessed on 22 November 2022)). 

Sequencing was performed on Illumina HiSeq2500 with single end reads of 150 base pairs at the CERBALAB Company (St. Petersburg, Russia, https://www.cerbalab.ru/, accessed on 22 November 2022). Illumina raw reads quality control was performed using fastqc program. Alignment of reads to the *V. vinifera* and *M. rotundifolia* genome assemblies was performed using BWA V. 0.7.17 (http://bio-bwa.sourceforge.net/ (accessed on 15 July 2022)) under default parameters.

The SNP calling procedure was performed using GATK v.4.2.6.0 software (https://github.com/broadinstitute/gatk/releases, accessed on 22 November 2022) after aligning reads on the reference genome assembly. For filtration of raw SNPs, VCFtools v. 0.1.16 (https://vcftools.github.io/downloads.html, accessed on 22 November 2022) was applied using the following parameters: minor allele frequency more than 2%, minimum depth 5, and minimum quality 30. 

The detected SNPs were filtered by number of missing data across the population with MISS = 0.75 and MISS = 0.90 thresholds. 

### 4.4. Linkage Mapping

The filtered SNPs (MISS = 0.90) were first checked for the segregation pattern expected for the heterozygous loci (1:2:1) using custom R script. 

Before linkage mapping, the SNPs were distributed into 20 linkage groups based on the alignment of the corresponding reads to the reference genome of *M. rotundifola* cv. Noble [7]. To do that, the genotype file was divided into chromosomes by converting from .csv to .vcf format using the TASSEL 5 v5.2.82 (https://bitbucket.org/tasseladmin/tassel-5-standalone/downloads/?tab=tags, accessed on 22 November 2022).

For mapping purposes, all segregating SNPs were first considered either homozygous (whether “hh” or “kk”) or heterozygous (“hk”) and were abbreviated as “AA” or “AB”, respectively. This made it possible to identify recombination events and estimate recombination fractions required for the ordering of SNP markers on the genetic map. 

Linkage analysis and marker re-ordering was performed using the Lep-MAP3 software [30] (https://sourceforge.net/p/lep-map3/wiki/LM3%20Home/, accessed on 22 November 2022). For offline work with all chromosomes, a bash script was written to perform the followed algorithm: 

ParentCall2 was used to call parental genotypes and chromosome markers.

Filtering2 was used to filter markers based on the segregation distortion. A *p*-value of 0.001 was used as threshold for the chi-square tests.

OrderMarkers2 orders the markers in the most likelihood position. To ensure that the order of markers is correct, the step was re-run multiple times with the reproducible result. 

Next, the marker order and pairwise recombination fractions were additionally checked with R/qtl software [31] (https://rqtl.org/, accessed on 22 November 2022) by using *est.rf* function, and then the Kosambi map function (*est.map*) was used for the final genetic distances estimation. 

Linkage phases between markers were estimated using the onemap::map function implemented in OneMap 2.8.2 software [14] (https://github.com/augusto-garcia/onemap, accessed on 28 October 2022). Option “Importing data from VCF file” (https://statgen-esalq.github.io/tutorials/onemap/Outcrossing_Populations.html, accessed on 28 October 2022) was used as recommended in the tutorials. The function estimates the multipoint log-likelihood, linkage phases and recombination frequencies for a sequence of markers in a given order. Otherwise, the best linkage phase combination is also estimated. The multipoint likelihood is calculated according to Wu et al. (2002) [13]. As the result, the parent and progeny phased haplotypes estimated by OneMap hidden Markov model (HMM) were generated (Supplementary Materials, Table S2).

### 4.5. QTL Mapping

For QTL analysis, the mapping population of eighty-four ‘Dixie’ S1 progenies with phased haplotypes (Supplementary Materials, Table S2) was employed. Each plant of the population was assessed for six morphological traits: average length of shoots, number of shoots, average leaf diameter, average internode length, number of internodes, and maximum diameter of shoots. 

Composite interval mapping (CIM) was performed through the package R/qtl using library(qtl) and cim function using EM algorithm. R/qtl recognized the input as F2 population. The Kosambi mapping function was used in the QTL analysis. Intervals of three background markers with a window width of 10 cM were analyzed to control the QTL background effect.

The LOD significance threshold was determined by a permutation test with 1000 permutations at a significance level of *p* < 0.05. The percentage of phenotype variation explained by the identified QTL were reported as suggested by “A Guide to QTL Mapping” [32].

## Figures and Tables

**Table 1:** Review of SNPs detected in the genome of *M. rotundifolia* cv. Dixie at different stages of the filtration procedure.

	SNPs Detected	Heterozygous cv. Dixie (%)	MISS = 0.75 ^1^	MISS = 0.90	chisq 75 ^2^	chisq 90
cv. Noble	1,783,373	21.6	114,301	34,904	18,412	3734
cv. Trayshed	578,975	17.9	20,380	5490	3997	n.d.
*V. vinifera*	2,127,067	19.3	95,965	31,490	n.d.	n.d.

^1^ MISS = 0.75—maximum missing calls is 75%. ^2^ chisq 75—SNPs with MISS = 0.75 passing chi-square goodness-of-fit test.

**Table 2:** Summary of genetic linkage map of *Muscadinia rotundifolia* cv. Dixie developed with SNP markers revealed using chromosome sequences of *M. rotundifolia* cv. Noble.

Linkage Group	No. of Markers	Total Distance (cM)	Average Distance (cM)	Maximum Distance (cM)
LG1	199	132.1	2.3	6.2
LG2	119	117.5	2.4	18.0
LG3	155	128.6	2.1	8.9
LG4	234	134.1	1.9	8.9
LG5	265	145.4	2.0	10.3
LG6	214	159.9	2.3	7.5
LG7	90	159.0	3.9	25.2
LG8	212	142.2	2.0	7.5
LG9	174	139.6	2.4	6.2
LG10	80	110.7	3.8	29.2
LG11	173	106.0	1.8	4.9
LG12	191	119.3	1.8	7.5
LG13	217	151.4	2.3	6.2
LG14	257	169.9	2.2	6.2
LG15	164	163.7	2.6	11.7
LG16	176	141.6	2.2	7.5
LG17	160	145.5	2.4	8.9
LG18	319	154.1	1.9	7.5
LG19	208	118.4	2.0	7.5
LG20	123	114.7	2.0	6.2
**Total**	**3730**	**2753.6**		

**Table 3:** Summary of morphological traits assessed for seedlings of cv. Dixie S1 population.

N	Morphological Trait	Mean ± sd	The Pearson Correlation Coefficient (r)				
			1	2	3	4	5
1	max. diameter of shoots, mm	3.78 ± 1.19					
2	number of shoots per plant, pcs	6.30 ± 3.15	0.496 ***				
3	average shoot length, cm	17.16 ± 11.1	0.500 ***	0.390 ***			
4	number of internodes, pcs	7.96 ± 2.87	0.566 ***	0.453 ***	0.899 ***		
5	average internode length, cm	1.98 ± 0.78	0.352 **	0.318 **	0.855 ***	0.609 ***	
6	average leaf diameter, cm	4.89 ± 1.23	0.470 ***	0.431 ***	0.680 ***	0.628 ***	0.701 ***

** *p*-value < 0.01, *** *p*-value < 0.001.

**Table 4:** Quantitative trait loci (QTL) mapped for morphological traits assessed for seedlings of cv. Dixie S1 population.

Trait	LG	Position	LOD	R^2^	SNP
average shoot length	14	150.55	5.94	27.8	14_24048713
average leaf diameter	14	150.55	4.12	20.2	14_24048713
average internode length	18	116.78	6.33	29.3	18_20602071 18_20601991 18_21074021 18_21074082 18_21141408

## Data Availability

The data presented in this study are available in this article and the Supplementary Materials.

## Supplementary Materials

The following supporting information can be downloaded at https://www.mdpi.com/article/10.3390/plants11233231/s1. Figure S1: The pedigree of *M. rotundifolia* cv. Dixie compiled by Prof. V. Nosulchak, Figure S2: MapCart of the linkage map of *M. rotundifolia* cv. Dixie, Figure S3: Chromosome-level distribution of SNP markers before and after passing the chi-square fit test, Figure S4: The source of fungal conidia to prepare inoculum for the experimental infection of the ‘Dixie’ S1 seedlings: (a) The grape bunches affected by oidium were collected in Livadia (the southern coast of Crimea); (b) leaves, infected by downy mildew collected in the Balaklava region of Crimea. Table S1: 3734 filtered SNPs subjected to linkage mapping, Table S2: The recombination pattern and linkage phases of 3734 SNPs on the genetic map of *M. rotundifolia* cv. Dixie.
