# Supplementary material for: Development of a High-Density Genetic Map for Muscadine Grape Using a Mapping Population from Selfing of the Perfect-Flowered Vine ‘Dixie’
Source: Plants (Basel). 2022 Nov 25;11(23):3231. doi: 10.3390/plants11233231 (PMC9738875; doi:10.3390/plants11233231)
Supplement: Supplementary file 1 [file plants-11-03231-s001.zip › SupplementaryData/Figure S2.pdf]

Supplementary Materials, Figure S2: MapCart of the linkage map of *M. rotundifolia* cv. Dixie

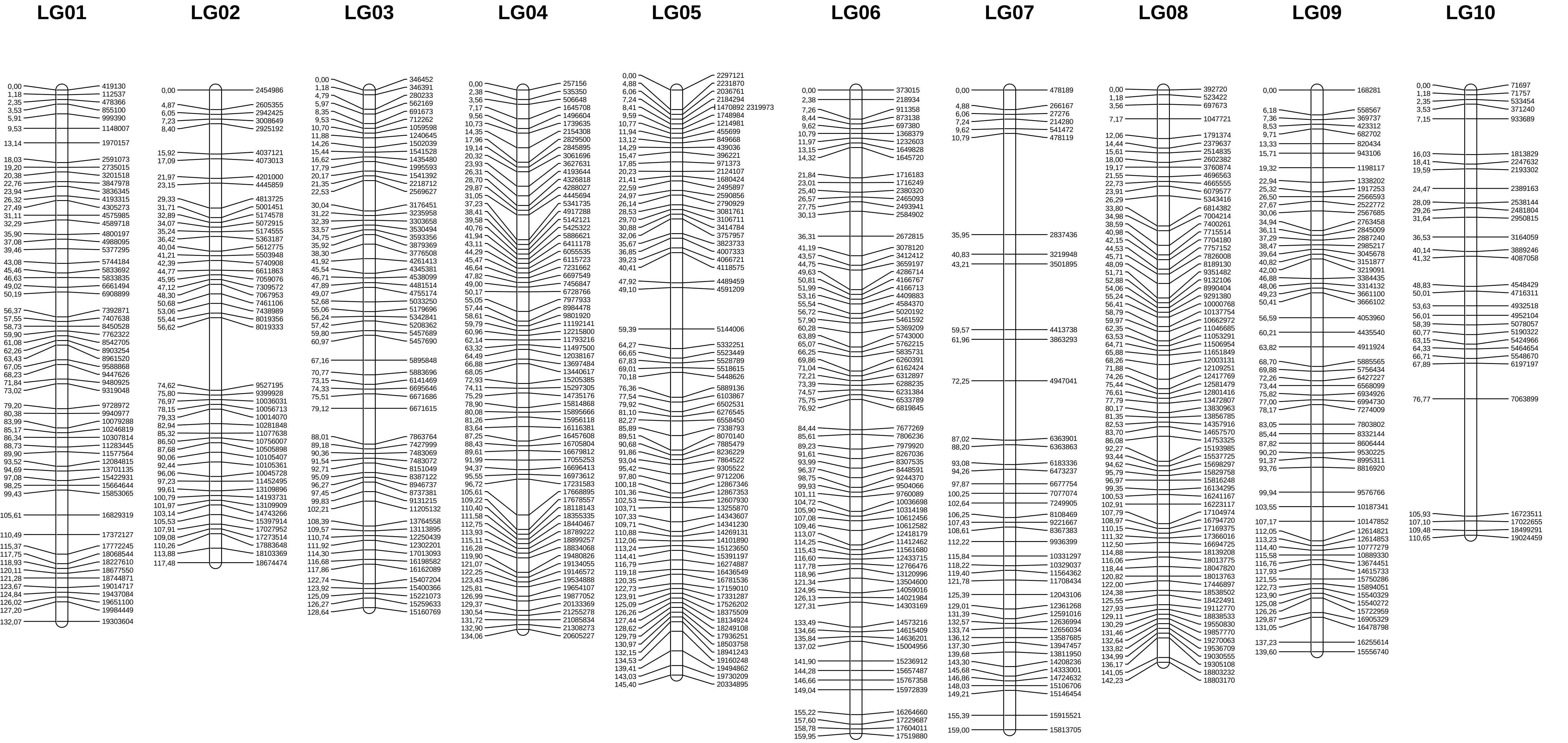

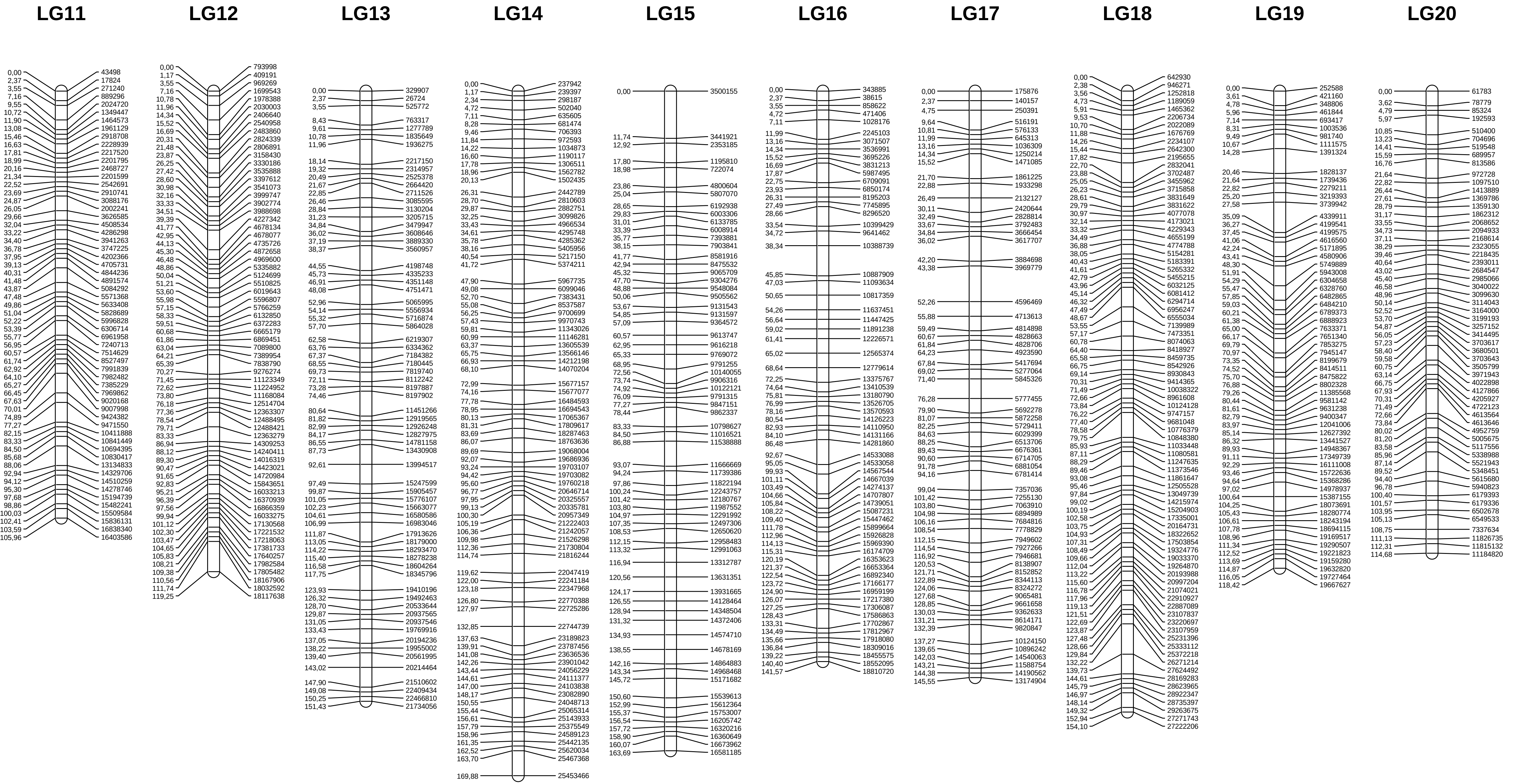
LG11
0,00 43498
2,37 17824
3,55 271240
7,16 889296
9,55 2024720
10,72 1349447
11,90 1464573
13,08 1961129
15,46 2918708
16,63 2228939
17,81 2217520
18,99 2201795
20,16 2468727
21,34 2201599
22,52 2542691
23,69 2910741
24,87 3088176
26,05 2002241
29,66 3626585
32,04 4508534
33,22 4286298
34,40 3941263
36,78 3747225
37,95 4202366
39,13 4705731
40,31 4844236
41,48 4891574
43,87 5084292
47,48 5571368
49,86 5633408
51,04 5828689
52,22 5996828
53,39 6306714
55,77 6961958
56,95 7240713
60,57 7514629
61,74 8527497
62,92 7991839
64,10 7982482
65,27 7385229
66,45 7969862
67,63 9020168
70,01 9007998
74,89 9424382
77,27 9471550
82,15 10411888
83,33 10841449
84,50 10694395
85,68 10830417
88,06 13134833
92,94 14329706
94,12 14510259
95,30 14278746
97,68 15194739
98,86 15482241
100,03 15509584
102,41 15836131
103,59 16838340
105,96 16403586
LG12
0,00 793998
1,17 409191
3,55 969269
7,16 1699543
10,78 1978388
11,96 2030003
14,34 2406640
15,52 2540958
16,69 2483860
20,31 2824339
21,48 2806891
23,87 3158430
26,25 3330186
27,42 3535888
28,60 3397612
30,98 3541073
32,16 3999747
33,33 3902774
34,51 3988698
39,39 4227342
41,77 4678134
42,95 4678077
44,13 4735726
45,30 4872658
46,48 4969600
48,86 5335882
50,04 5124699
51,21 5510825
53,60 6019643
55,98 5596807
57,15 5766259
58,33 6132850
59,51 6372283
60,68 6665179
61,86 6869451
63,04 7089800
64,21 7389954
65,39 7838790
70,27 9276274
71,45 11123349
72,62 11224952
73,80 11168084
76,18 12514704
77,36 12363307
78,54 12488495
79,71 12488421
83,33 12363279
86,94 14309253
88,12 14240411
89,30 14016319
90,47 14423021
91,65 14720984
92,83 15843651
95,21 16033213
96,39 16370959
97,56 16866359
99,94 16033275
101,12 17130568
102,30 17221532
103,47 17218063
104,65 17381733
105,83 17640257
108,21 17982584
109,38 17805482
110,56 18167906
111,74 18032592
119,25 18117638
LG13
0,00 329907
2,37 26724
3,55 525772
8,43 763317
9,61 1277789
10,78 1835649
11,96 1936275
18,14 2217150
19,32 2314957
20,49 2525378
21,67 2664420
22,85 2711526
26,46 3085595
28,84 3130204
31,23 3205715
34,84 3479947
36,02 3608646
37,19 3889330
38,37 3560957
44,55 4198748
45,73 4335233
46,91 4351148
48,08 4751471
52,96 5065995
54,14 5556934
55,32 5716874
57,70 5864028
62,58 6219307
63,76 6334362
67,37 7184382
68,55 7180445
69,73 7819740
72,11 8112242
73,28 8197887
74,46 8197902
80,64 11451266
81,82 12919565
82,99 12926248
84,17 12827975
86,55 14781158
87,73 13430908
92,61 13994517
97,49 15247599
99,87 15905457
101,05 15776107
102,23 15663077
104,61 16580586
106,99 16983046
111,87 17913626
113,05 18179000
114,22 18293470
115,40 18278238
116,58 18604264
117,75 18345796
123,93 19410196
126,32 19492463
128,70 20533644
129,87 20937565
131,05 20937546
133,43 19769916
137,05 20194236
138,22 19955002
139,40 20561995
143,02 20214464
147,90 21510602
149,08 22409434
150,25 22466810
151,43 21734056
LG14
0,00 237942
1,17 239397
2,34 298187
4,72 502040
7,11 635605
8,28 681474
9,46 706393
11,84 972593
14,22 1034873
16,60 1190117
17,78 1306511
18,96 1562782
20,13 1502435
26,31 2442789
28,70 2810603
29,87 2882751
32,25 3099826
33,43 4966534
34,61 4295748
35,78 4285362
38,16 5405956
40,54 5217150
41,72 5374211
47,90 5967735
49,08 6099046
52,70 7383431
55,08 8537587
56,25 9700699
57,43 9970743
59,81 11343026
60,99 11146281
63,37 13605539
65,75 13566146
66,93 14212198
68,10 14070204
72,99 15677157
74,16 15677077
77,78 16484593
78,95 16694543
80,13 17065367
81,31 17809617
83,69 18287463
86,07 18763636
89,69 19068004
92,07 19686936
93,24 19703107
94,42 19703082
95,60 19760218
96,77 20646714
97,95 20325557
99,13 20335781
100,30 20957349
105,19 21222403
106,36 21242057
109,98 21526298
112,36 21730804
114,74 21816244
119,62 22047419
122,00 22241184
123,18 22347968
126,80 22770388
127,97 22725286
132,85 22744739
137,63 23189823
139,91 23787456
141,08 23636536
142,26 23901042
143,44 24056229
144,61 24111377
147,00 24103838
148,17 23082890
150,55 24048713
155,44 25065314
156,61 25143933
157,79 25375549
158,96 24589123
161,35 25442135
162,52 25620034
163,70 25467368
169,88 25453466
LG15
0,00 3500155
11,74 3441921
12,92 2353185
17,80 1195810
18,98 722074
23,86 4800604
25,04 5807070
28,65 6192938
29,83 6003306
31,01 6133785
33,39 6008914
35,77 7393881
38,15 7903841
41,77 8581916
42,94 8475532
45,32 9065709
47,70 9304276
48,88 9548084
50,06 9505562
53,67 9131543
54,85 9131597
57,09 9364572
60,57 9613747
62,95 9616218
65,33 9769072
68,95 9791255
72,56 10140055
73,74 9906316
74,92 10122121
76,09 9791315
77,27 9847151
78,44 9862337
83,33 10798627
84,50 11016521
86,88 11538888
93,07 11666669
94,24 11739386
97,86 11822194
100,24 12243757
101,42 12180767
103,80 11987552
104,97 12291992
107,35 12497306
108,53 12650620
112,15 12958483
113,32 12991063
116,94 13312787
120,56 13631351
124,17 13931665
126,55 14128464
128,94 14348504
131,32 14372406
134,93 14574710
138,55 14678169
142,16 14864883
143,34 14968468
145,72 15171682
150,60 15539613
152,99 15612364
155,37 15753007
156,54 16205742
157,72 16320216
158,90 16360649
160,07 16673962
163,69 16581185
LG16
0,00 343885
2,37 38615
3,55 858622
4,72 471406
7,11 1028176
11,99 2245103
13,16 3071507
14,34 3536991
15,52 3695226
16,69 3831213
17,87 5987495
22,75 6709091
23,93 6850174
26,31 8195203
27,49 7745895
28,66 8296520
33,54 10399429
34,72 9641462
38,34 10388739
45,85 10887909
47,03 11093634
50,65 10817359
54,26 11637451
56,64 11447425
59,02 11891238
61,41 12226571
65,02 12565374
68,64 12779614
72,25 13375767
74,64 13410539
75,81 13180790
76,99 13526705
78,16 13570593
80,54 14126223
82,93 14110950
84,10 14131166
86,48 14281860
92,67 14533088
95,05 14533058
99,93 14567544
101,11 14667039
103,49 14274137
104,66 14707807
105,84 14739051
108,22 15087231
109,40 15447462
111,78 15899664
112,96 15926828
114,13 15969390
115,31 16174709
120,19 16353623
121,37 16653364
122,54 16892340
123,72 17166177
124,90 16959199
126,07 17217380
127,25 17306087
128,43 17586863
133,31 17702867
134,49 17812967
135,66 17918080
136,84 18309016
139,22 18455575
140,40 18552095
141,57 18810720
LG17
0,00 175876
2,37 140157
4,75 250391
9,64 516191
10,81 576133
11,99 645313
13,16 1036309
14,34 1250214
15,52 1471085
21,70 1861225
22,88 1933298
26,49 2132127
30,11 2420644
32,49 2828814
33,67 3792483
34,84 3666454
36,02 3617707
42,20 3884698
43,38 3969779
52,26 4596469
55,88 4713613
59,49 4814898
60,67 4828663
61,84 4828706
64,23 4923590
67,84 5417694
69,02 5277064
71,40 5845326
76,28 5777455
79,90 5692278
81,07 5872258
82,25 5729411
84,63 6029399
88,25 6513706
89,43 6676361
90,60 6714705
91,78 6881054
94,16 6781414
99,04 7357036
101,42 7255130
103,80 7063910
104,98 6894989
106,16 7684816
108,54 7778829
112,15 7949602
114,54 7927266
116,92 7946681
120,53 8138907
121,71 8152852
122,89 8344113
124,06 8324272
127,68 9065481
128,85 9661658
130,03 9362633
131,21 8614171
132,39 9820847
137,27 10124150
139,65 10896242
142,03 14540063
143,21 11588754
144,38 14190562
145,55 13174904
LG18
0,00 642930
2,38 946271
3,56 1252818
4,73 1189059
5,91 1465362
9,53 2206734
10,70 2022089
11,88 1676769
14,26 2234107
15,44 2642300
17,82 2195655
22,70 2832041
23,88 3702487
25,05 3455962
26,23 3715858
28,61 3831649
29,79 3831622
30,97 4077078
32,14 4173021
33,32 4229343
34,49 4655199
36,88 4774788
38,05 5154281
40,43 5183391
41,61 5265332
42,79 5455215
43,96 6032125
45,14 6081412
46,32 6294714
47,49 6956247
48,67 6555034
53,55 7139989
57,17 7473351
60,78 8074063
64,40 8418927
65,58 8459735
66,75 8542926
69,14 8930843
70,31 9414365
71,49 10038322
72,66 8961608
73,84 10124128
76,22 9747157
77,40 9681048
78,58 10776379
79,75 10848380
85,93 11033448
87,11 11080581
88,29 11247635
89,46 11373546
93,08 11861647
95,46 12505528
97,84 13049739
99,02 14215974
100,19 15204903
102,58 17335001
103,75 20164731
104,93 18322652
107,31 17503854
108,49 19324776
109,66 19033370
112,04 19264870
113,22 20193988
115,60 20997204
116,78 21074021
117,96 22910927
119,13 22887089
121,51 23107837
122,69 23220697
123,87 23107959
127,48 25231396
128,66 25333112
129,84 25372218
132,22 26271214
139,73 27624492
144,61 28169283
145,79 28623965
146,97 28922347
148,14 28735397
149,32 29263675
152,94 27271743
154,10 27222206
LG19
0,00 252588
3,61 421160
4,78 348806
5,96 461844
7,14 693417
8,31 1003536
9,49 981740
10,67 1111575
14,28 1391324
20,46 1828137
21,64 1739436
22,82 2279211
25,20 3219393
27,58 3739942
35,09 4339911
36,27 4199541
37,45 4199575
41,06 4616560
42,24 5171895
43,41 4580906
48,30 5749889
51,91 5943008
54,29 6304658
55,47 6328760
57,85 6482865
59,03 6484210
60,21 6789373
61,38 6888923
65,00 7633371
66,17 7651340
69,79 7853275
70,97 7945147
73,35 8199679
74,52 8414511
75,70 8475822
76,88 8802328
79,26 11385568
80,44 9581142
81,61 9631238
82,79 9400347
83,97 12041006
85,14 12627392
86,32 13441527
89,93 14948367
91,11 17349739
92,29 16111008
93,46 15722636
94,64 15368286
97,02 14978937
100,64 15387155
104,25 18073691
105,43 18280774
106,61 18243194
107,78 18694115
108,96 19169517
111,13 19290507
112,52 19221823
113,69 19159280
114,87 19632820
116,05 19727464
118,42 19667627
LG20
0,00 61783
3,62 78779
4,79 85324
5,97 192593
10,85 510400
13,23 704696
14,41 519548
15,59 689957
16,76 813586
21,64 972728
22,82 1097510
26,44 1413889
27,61 1369786
28,79 1359130
31,17 1862312
33,55 2068652
34,73 2094933
37,11 2168614
38,29 2323055
39,46 2218435
40,64 2393011
43,02 2684547
45,40 2985066
46,58 3040022
48,96 3099630
50,14 3114043
52,52 3164000
53,70 3199193
54,87 3257152
56,05 3414495
57,23 3703617
58,40 3680501
59,58 3703643
60,75 3505799
63,14 3971943
66,75 4022898
67,93 4127866
70,31 4205927
71,49 4722123
72,66 4613564
73,84 4613646
80,02 4952759
81,20 5005675
83,58 5117556
85,96 5338988
87,14 5521943
89,52 5348451
94,40 5615680
96,78 5940823
100,40 6179393
101,57 6179336
103,95 6502678
105,13 6549533
108,75 7337634
111,13 11826735
112,31 11815132
114,68 11184820
