# Supplementary material for: Development of a High-Density Genetic Map for Muscadine Grape Using a Mapping Population from Selfing of the Perfect-Flowered Vine ‘Dixie’
Source: Plants (Basel). 2022 Nov 25;11(23):3231. doi: 10.3390/plants11233231 (PMC9738875; doi:10.3390/plants11233231)
Supplement: Supplementary file 1 [file plants-11-03231-s001.zip › SupplementaryData/Figure S3.pdf]

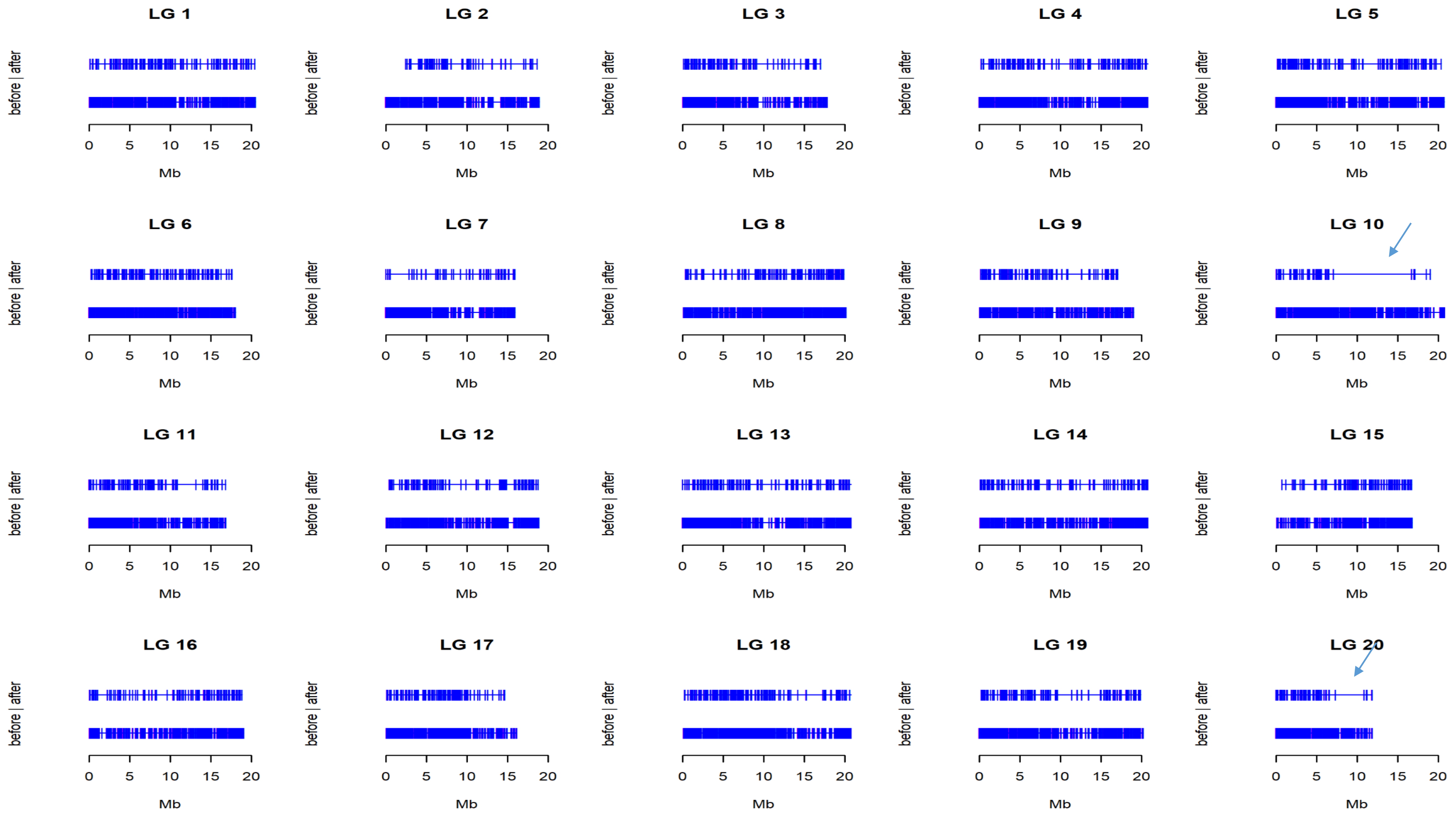

Supplementary Materials, Figure S3. Chromosome-level distribution of SNP markers **before** and **after** passing the chi-square fit test
