# Supplementary material for: Development of a High-Density Genetic Map for Muscadine Grape Using a Mapping Population from Selfing of the Perfect-Flowered Vine ‘Dixie’
Source: Plants (Basel). 2022 Nov 25;11(23):3231. doi: 10.3390/plants11233231 (PMC9738875; doi:10.3390/plants11233231)
Supplement: Supplementary file 1 [file plants-11-03231-s001.zip › SupplementaryData/Figure S4.pdf]

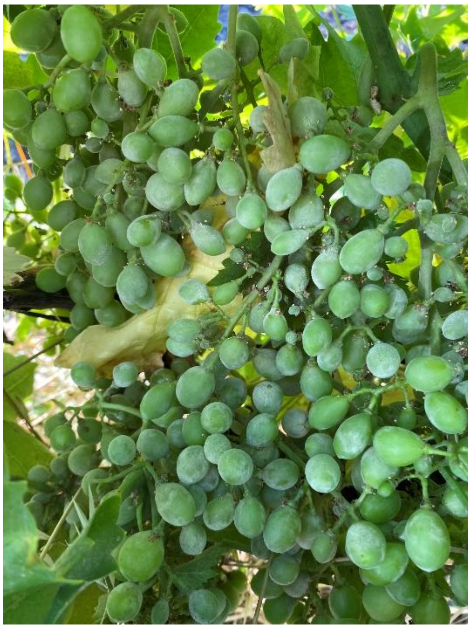

(**a**)

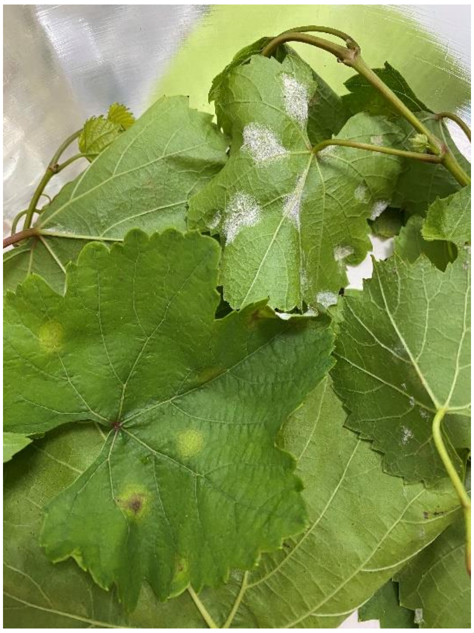

(**b**)

Supplementary Materials, Figure S3. The source of fungal conidia to prepare inoculum for the experimental infection of the ‘Dixie’ S1 seedlings: (**a**) The grape bunches affected by odium were collected in Livadia (the southern coast of Crimea); (**b**) leaves, infected by downy mildew collected in the Balaklava region of Crimea.
