# Supplementary figures and images for: Development of a High-Density Genetic Map for Muscadine Grape Using a Mapping Population from Selfing of the Perfect-Flowered Vine ‘Dixie’
Source: Plants (Basel). 2022 Nov 25;11(23):3231. doi: 10.3390/plants11233231 (PMC9738875; doi:10.3390/plants11233231)

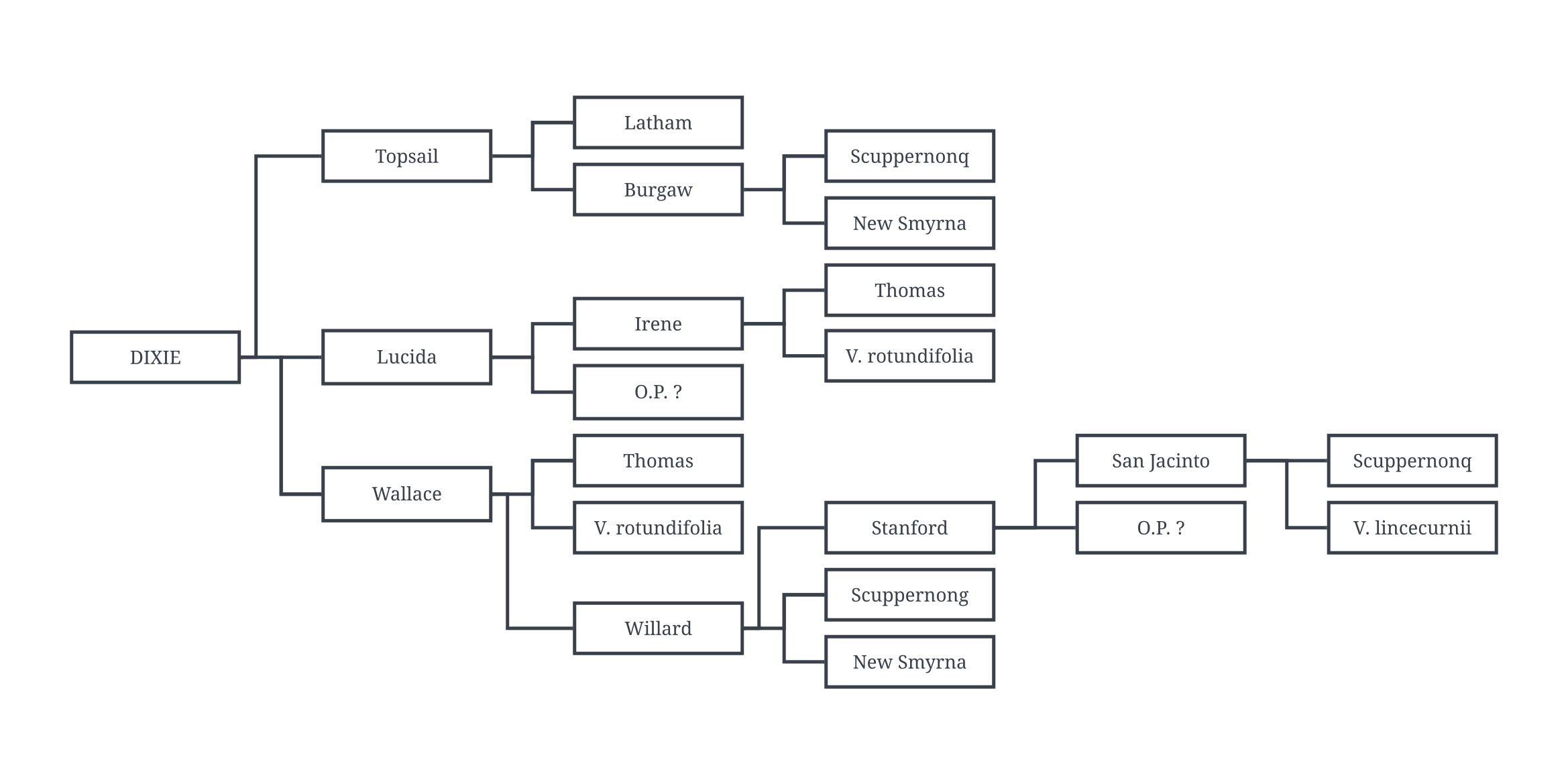

Supplement: Supplementary file 1 [file plants-11-03231-s001.zip › SupplementaryData/Figure S1.tiff]
